# The catabolic-to-anabolic shift seen in the canine osteoarthritic cartilage treated with knee joint distraction occurs after the distraction period

**DOI:** 10.1016/j.jot.2022.09.003

**Published:** 2022-10-20

**Authors:** M. Teunissen, B.P. Meij, L. Snel, K. Coeleveld, J. Popov-Celeketic, I.S. Ludwig, F. Broere, F.P.J.G. Lafeber, M.A. Tryfonidou, S.C. Mastbergen

**Affiliations:** a Department of Clinical Sciences, Faculty of Veterinary Medicine, Utrecht University, Utrecht the Netherlands; b Rheumatology & Clinical Immunology, UMC Utrecht, Utrecht University, Utrecht the Netherlands; c Department of Biomolecular Health Sciences, Faculty of Veterinary Medicine, Utrecht University, Utrecht the Netherlands

**Keywords:** Canine, Knee joint distraction, Mechanical loading, Proteoglycans, TGF-β signalling

## Abstract

**Background:**

Cartilage regenerative mechanisms initiated by knee joint distraction (KJD) remain elusive. Animal experiments that are representative for the human osteoarthritic situation and investigate the effects of KJD at consecutive time points could be helpful in this respect but are lacking. This study investigated the effects of KJD on the osteoarthritic joint of dogs on two consecutive timepoints.

**Methods:**

Osteoarthritis was bilaterally induced for 10 weeks in 12 dogs using the groove model. Subsequently, KJD was applied to the right hindlimb for 8 weeks. The cartilage, subchondral bone and synovial membrane were investigated directly after KJD treatment, and after 10 weeks of follow-up after KJD treatment. Macroscopic and microscopic joint tissue alterations were investigated using the OARSI grading system. Additionally, proteoglycan content and synthesis of the cartilage were assessed biochemically. RT-qPCR analysis was used to explore involved signaling pathways.

**Results:**

Directly after KJD proteoglycan and collagen type II content were reduced accompanied by decreased proteoglycan synthesis. After 10 weeks of follow-up, proteoglycan and collagen type II content were partly restored and proteoglycan synthesis increased. RT-qPCR analysis of the cartilage suggests involvement of the TGF-β and Notch signalling pathways. Additionally, increased subchondral bone remodelling was found at 10 weeks of follow-up.

**Conclusion:**

While the catabolic environment in the cartilage is still present directly after KJD, at 10 weeks of follow-up a switch towards a more anabolic joint environment was observed. Further investigation of this timepoint and the pathways involved might elucidate the regenerative mechanisms behind KJD.

**The Translational Potential of this Article:**

Further elucidation of the regenerative mechanisms behind KJD could improve the existing KJD treatment. Furthermore, these findings could provide input for the discovery or improvement of other joint regenerative treatment strategies.

## 1 Introduction

Knee joint distraction (KJD) is a joint preservative treatment strategy that may delay or prevent knee arthroplasty in end-stage osteoarthritis (OA) [1]. During KJD, the joint is temporarily distracted using an external fixation frame with built-in springs, while weightbearing of the leg is encouraged to allow for intermittent joint fluid pressure changes. Limited clinical studies, with relatively small sample size, provide evidence that KJD causes prolonged clinical benefit and structural improvement [1]. At one and two years of follow-up after KJD, an increased radiographic (minimum) joint space width and increased cartilage thickness on MRI were found [2]. Both the clinical and structural improvement after KJD were prolonged over time, as 48% of patients treated with KJD were not treated with total knee arthroplasty at 10 years of follow-up, with lasting cartilage changes observed by MRI [3,4]. Furthermore, at two years of follow-up, the net collagen type II (COL2) synthesis was increased [2]. Although these findings suggest tissue regeneration, direct evidence of cartilage tissue repair can only be conducted using animal studies.

KJD induced ingrowth of cartilage-like tissue in osteochondral defects in rabbits [[5], [6], [7]] and attenuated cartilage degradation and subchondral bone changes in rats with anterior cruciate ligament transection (ACLT)-induced OA [8]. However, small animal models do not resemble the human OA situation well [9]. KJD was mostly applied directly after or shortly after inflicting cartilage damage [5,6], incomparable to the gradual onset of OA in humans and KJD applied in joints with fully established OA. Furthermore, the natural repair activity of small mammals is much higher compared to humans [9], and specifically in rabbits joint loading is very different [9]. Within this context, dogs are a valuable alternative to study cartilage repair as they lack significant intrinsic ability to heal cartilage defects, similar to humans, and canine OA bears a close resemblance to human OA [10]. In a canine ACLT model, with 16 weeks of OA establishment prior to KJD treatment, synovitis decreased and the proteoglycan (PG) turnover normalized directly after 8 weeks of KJD, although no evidence for cartilage repair was identified [11]. In a canine groove model, 25 weeks follow-up after KJD, improved macroscopic and histologic damage scores, higher proteoglycan content, better retention of newly formed PG and less collagen damage were reported in KJD-treated joints compared to the OA controls [12]. It was therefore hypothesized that tissue structure modification is initiated during distraction and proceeds in the post-distraction period, resulting in the tissue repair seen in human clinical studies. However, a recent study, employing KJD 10 weeks after OA was induced with the groove model, showed that midway KJD treatment (after 4 weeks of KJD), cartilage integrity decreased [13]. This was reflected by increased histological OARSI score and upregulation of catabolic genes in the distracted joint as compared to OA joint [13].

Up until now, comprehensive animal experiments that investigate the progression of the effects of KJD at consecutive time points within one relevant animal model are lacking. Based on earlier canine studies we hypothesized that the shift towards repair activity occurs either at the end of the KJD period or in the subsequent follow-up period. Therefore, in the present study, a bilateral OA groove was employed to investigated joint tissues directly after 8 weeks of unilateral KJD, and at 10 weeks of follow-up after KJD. Combined with the studies performed in the same canine groove model of OA at an earlier and later timepoint of follow up [12,13], this explorative study provides complementary insights into the regenerative mechanisms behind KJD.

## 2 Methods and materials

### 2.1 Animals and experimental procedures

Skeletally mature mixed-breed dogs (n ​= ​12, females, 16.1 ​± ​5.1 (mean ​± ​SD) months and 20.9 ​± ​2.6 ​kg) were obtained from Marshall BioResources (North Rose, NY, USA) after ethical approval (AVD1080020173964). Extensive information about the animal husbandry and experimental procedures is described in supplementary file 1.1 and 1.2. Sample size calculation used the size effects obtained in the previous KJD study [13].

**Fig. 1:**
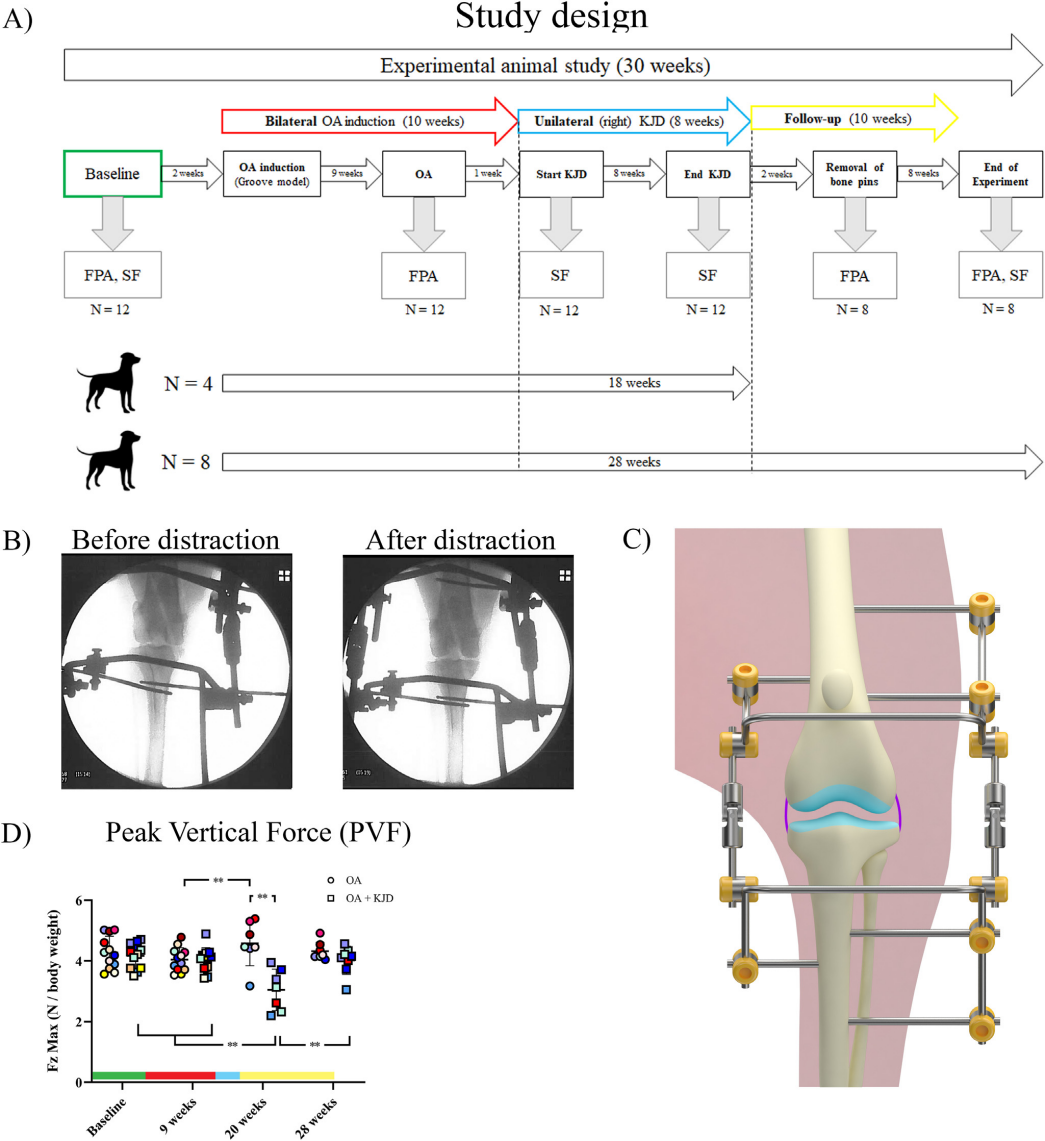
**Study design of the experimental animal study (A).** Force plate analysis (FPA) and Synovial Fluid (SF) biopsy was performed at baseline in all dogs (N ​= ​amount of dogs used). After 2 weeks, osteoarthritis (OA) was induced bilateral according to the groove model in all dogs (N ​= ​12). At the end of the OA induction (at 9 weeks), FPA was performed. After 10 weeks of OA induction, knee joint distraction (KJD) was applied to the right hindlimb for 8 weeks. At the start and end of KJD, SF was collected. Directly after KJD (at week 18 of the experimental study), 4 dogs were euthanized. Of the other 8 dogs, the frame was removed. After 2 weeks, the bone pins were removed and FPA was performed. After 8 more weeks (at week 28 of the experimental study), FPA and SF biopsies were performed and the dogs were euthanized. **Fluoroscopic images of application of distraction (B)** were taken during surgery just before distraction and directly after the application of distraction to determine the sufficiency of the amount of distraction. **Schematic image of the distraction frame (C)** showing the placement of the bone pins and their connection with the external frame. The tibial and femoral part of the frame are connected by hinges, allowing for the ability to flex and extend the knee joint. **The peak vertical force (PVF) (D)**, also known as the maximal vertical force (Fz max) (in newton (N) corrected for body weight), was determined in both hindlimbs at baseline (before the induction of OA), at 9 weeks (after 9 weeks of OA induction), at 20 weeks (after the bone pins were removed), and at 28 weeks (after 10 weeks of follow-up). The round dots represent the left hindlimb, which, after week 10, represents the OA control limb (OA). The squared dots represent the right hindlimb, which, after week 10, represents the distracted hindlimb (OA ​+ ​KJD). The individual colours represent individual donors. ∗∗p ​< ​0.01. The bars represent the different phases of the experimental study; green ​= ​baseline (healthy), red ​= ​OA induction (10 weeks), blue ​= ​KJD treatment (8 weeks), yellow ​= ​Follow-up (10 weeks). (For interpretation of the references to colour in this figure legend, the reader is referred to the Web version of this article.)

Knee OA was induced bilaterally according to the canine groove model, as described before [14]. After 10 weeks of OA induction, distraction of the right knee joint was applied for 8 weeks employing an external fixation frame with a hinge bridging the joint [12]. Joint distraction was achieved, intra-operatively visualized using fluoroscopy (Fig. 1B), maintaining smooth motion of the joint during flexion and extension. The extend of distraction and integrity of the bone pins were monitored every two weeks using radiography and adjusted if necessary.

Dogs were randomly divided in two groups. The first group (n ​= ​4) was euthanized directly after the end of the 8-week KJD procedure. At the same timepoint, in the second group KJD was ended by removing the connecting rods and hinges to force the dogs to gradually reload the joint. The fixation frames and bone pins were removed two weeks later (Fig. 1A). The second group (n ​= ​8) was euthanized after a 10-week follow-up period post-KJD.

### 2.2 Gait analysis

Limb loading was assessed using gait analysis (Fig. 1A) as described previously [15]. Briefly, vertical (Fz) and craniocaudal (Fy) forces at walking speed were measured using a force plate mounted flush with the surface of an 11-meter walkway with 100 ​Hz sampling frequency. The average of 10 successful recordings per limb (one measurement) was normalized to body weight and expressed in N/kg. For each timepoint, two to three measurements were performed on consecutive days.

### 2.3 Processing of the material

After euthanasia, both hindlimbs were processed within 2 ​h. High resolution photographs of the joint were obtained. Cartilage from the weight-bearing area of the femoral condyles and tibial plateaus, and patellar synovium were collected and fixed in 4% phosphate-buffered formalin containing 2% sucrose (pH 7.0) for (immuno)histochemistry or snap frozen for RNA isolation. Additionally, cartilage was collected for biochemical analysis. Subchondral bone samples were collected from the femoral condyles and tibial plateaus after the cartilage was collected, resulting in an osteochondral bone piece with a variable layer of cartilage. Subsequently, subchondral bone samples were fixed in 4% neutral buffered formaldehyde and decalcified using 0.5 ​M EDTA (pH 7.0; 108,421, Merck).

### 2.4 **Macroscopic, microscopic and biochemical outcome measur**es

Macroscopic scoring of cartilage damage and synovial inflammation, and microscopic scoring of cartilage, synovium and subchondral bone alterations were performed randomized and blind according to the Osteoarthritis Research Society International (OARSI) canine scoring system [16] by three observers to determine the average OARSI score for each parameter. Cartilage and subchondral bone sections were stained with Safranin-O/Fast green. Synovium sections were stained with Hematoxylin/Eosin (HE). Furthermore, the cartilage matrix was evaluated for the immunopositivity of collagen type-1 (COL1A1), and −2 (COL2A1) (Supplementary file 1.3.1). Proteoglycan synthesis was determined using ^35^SO_4_^2−^, as described previously [12] (Supplementary file 1.3.2). Briefly, after 4 ​h of labeling with Na_2_^35^SO_4_ (NEX-041-H, carrier free, Dupont) and a subsequent 3-day culture period, cartilage was digested and labelled proteoglycans were precipitated using cetylpyridium chloride (CPC; Sigma C-9002) measured by liquid scintillation analysis (TriCarb, Perkin Elmer). Proteoglycans were precipitated and stained by Alcian Blue (Sigma A5268) in either tissue digest or culture medium, to determine the proteoglycan content or release. The staining was quantified spectrophotometrically according to change in absorbance at 620 ​nm. Chondroitin sulphate (Sigma C4383) was used as a reference.

For Alkaline phosphatase (ALP) activity, homogenized subchondral bone powder was dissolved in Tris–HCl+2% Triton (1:1) solution and ALP was measured using a p-nitrophenyl phosphate assay and normalized for DNA content measured using the Qubit™ dsDNA BR Assay. (Supplementary file 1.3.4).

### 2.5 Gene expression analysis by RT-qPCR

A mikro-dismembrator (B. Braun Biotech International) was used to reduce snap-frozen cartilage samples and subchondral bone plugs to powder (two cycles; 2000 ​rpm). After lysis with QIAzol Lysis Reagent (79,306, Qiagen) total RNA was extracted and quantified as described previously [13] (Supplementary file 1.3.3).

Quantitative RT-PCR was performed using IQ SYBR Green SuperMix (Biorad) and a CFX384 Touch™ Real-Time PCR Detection System (Biorad). 7 reference genes were employed: *RPS19, SDHA, YWHAZ, TBP, RPS5, RPL13 and HPRT*. When the average Cq of the references genes was higher than 35 samples were excluded considering them of insufficient quality. Relative gene expression was calculated using the Livak method (2^^−ΔΔCq^).

### 2.6 Synovial fluid analysis

Synovial fluid (SF) was aseptically collected from both stifle joints at baseline, at 10 weeks after OA induction, at 18 and 28 weeks (Fig. 1A). The SF was kept on ice and immediately processed by centrifugation (5 ​min, 500 ​g, 4 ​°C) to remove cellular debris, aliquoted and stored at −80 ​°C until further processing. SF samples were treated with hyaluronidase (1:1, final concentration 4 ​mg/ml, Sigma) and subjected to a canine multiplex ELISA (CCYTOMAG-90 ​K, Merck) to measure interleukin (IL)-6, −8, −10, −15, −18 and C–C Motif Chemokine Ligand 2 (CCL2) according to the manufacturer's protocol on a Luminex analyser (MAGPIX®, Luminex Corporation). TGFβ1 was measured in acid activated SF, without hyaluronidase treatment, using the TGF-beta 1 Quantikine ELISA Kit (MB100B, R&D Systems, Inc.) according to the manufacturer's protocol. +

### 2.7 Statistical analysis

Statistical analysis was performed using R Statistics (R version 3.6.3, RStudio version 1.2.5033) (Supplementary file 1.4). For normally distributed data, linear mixed models were employed. If the data was not normally distributed, a Kruskal–Wallis test and Dunn's Multiple Comparison Test were used. P values were subjected to corrections for multiple testing (Benjamini-Hochberg False Discovery Rate) if applicable (Supplementary file 1.4.). Effect sizes (ES) with 95% confident intervals (CI) were calculated using Hedge's g (HG) for normally distributed data and Cliff's delta (CD) for non-normally distributed data. Because of the explorative nature of the study, outcomes with a *p* value of <0.05 or *p* ​< ​0.15 in combination with a large effect size (HG ​> ​0.8, CD ​> ​0.47) were considered relevant for discussion.

## 3 Results

### 3.1 Animals

During the OA induction phase, all dogs were fully active with subjectively normal joint loading and movement. Minor adverse effects were observed that resolved with appropriate treatment (Supplementary file 2).

### 3.2 Gait analysis

Directly after KJD, the peak vertical force (PVF) of OA ​+ ​KJD-limbs was significantly decreased compared to OA-limbs (*p* ​< ​0.001), and compared to all other time points in OA ​+ ​KJD-limbs (*p* ​< ​0.001) (Fig. 1C). In OA-limbs the PVF increased directly after KJD compared to 9 weeks (*p* ​= ​0.02) suggesting a load shift directly after KJD treatment to compensate for the decreased loading of the OA ​+ ​KJD-limb. This shift was not detected after follow-up.

### 3.3 Macroscopic assessment of cartilage damage and synovial inflammation

**Fig. 2:**
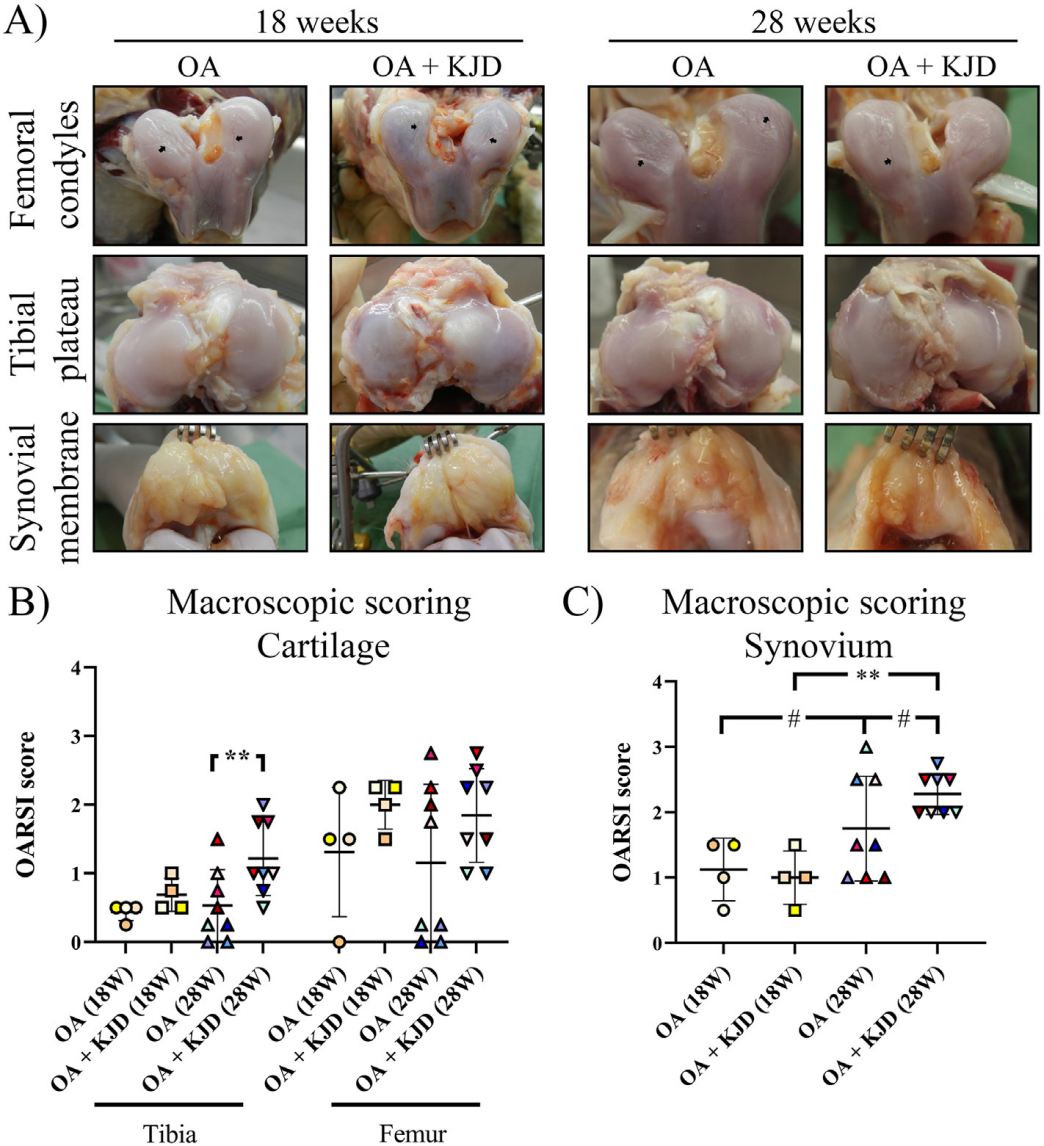
**Macroscopic evaluation of cartilage damage and synovitis (A)** High resolution photographs were obtained of the femoral condyles, tibial plateaus and synovium of the osteoarthritic (OA) control joint and the distracted joint (OA ​+ ​KJD) at 18 and 28 weeks. The grooves applied to induce OA are visible (black arrows). The average **OARSI scoring of cartilage damage (B) and synovitis (C)**, scored by two observers, showed a mild score for all conditions. Individual coloured dots represent individual donors. ∗∗p ​< ​0.01; # 0.15 ​> ​p ​> ​0.05 with a large ES.

Directly after KJD, there were no differences in macroscopic cartilage damage between OA ​+ ​KJD joints and OA controls (Fig. 2A and B). After follow-up, the OARSI score of OA ​+ ​KJD joints was higher compared to OA controls (*p* ​= ​0.009; ES (CD): 0.67) in the tibia. During tissue collection, we observed that in the OA control joints of 5/12 animals, the grooves were situated in the patellar groove instead of on the weight bearing surface of the femoral condyles (OARSI score of 0 (Fig. 2B)). A sensitivity assay (Supplementary file 2.1) demonstrated that these joints behaved in similar fashion to all other OA joints and the data was included in this and further analysis.

Macroscopic synovial inflammation was generally low directly after KJD in both joints (Fig. 2A and C) and increased at follow-up in the OA controls (*p* ​= ​0.09; ES (HG): 0.8). At follow-up, in OA ​+ ​KJD joints synovial inflammation increased compared to OA ​+ ​KJD joints directly after KJD (*p* ​= ​0.001; ES (HG): 3.44) and to OA joints at follow-up (*p* ​= ​0.07; ES (HG): 0.83).

### 3.4 Histological evaluation of cartilage alterations

**Fig. 3:**
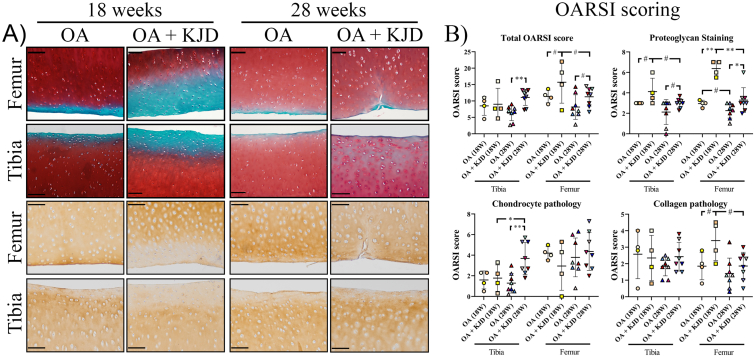
**Microscopic scoring of cartilage alterations (A)** Histological evaluation of the cartilage was performed at 18 and 28 weeks for the osteoarthritic (OA), and OA treated with knee joint distraction (OA ​+ ​KJD) joints. Representative images of the average total OARSI score are shown for the tibia and femur, scored by three observers. The OARSI subscores; Proteoglycan Staining and Chondrocyte Pathology were evaluated using Safranin-O/Fast green staining (upper two panels). The Collagen Pathology was scored using a collagen type II (COL2) immunohistochemical staining (lower two panels). Scale bar is set at 100 ​μM **(B) Individual OARSI Subscores** (Proteoglycan staining, Chondrocyte pathology, Cartilage surface (not displayed), and Collagen pathology) were scored blind according to the OARSI scoring system. Subsequently, the total sum of the individual subscores was shown as the Total OARSI score. Individual coloured dots represent individual donors. ∗∗p ​< ​0.01; ∗p ​< ​0.05; # 0.15 ​> ​p ​> ​0.05 with a large ES. (For interpretation of the references to colour in this figure legend, the reader is referred to the Web version of this article.)

Directly after KJD in the femur and tibia there was reduced safranin O intensity, indicating loss of PG, and reduced COL2 immunopositivity in OA ​+ ​KJD joints compared to OA controls (Fig. 3A). The total OARSI score increased in the OA ​+ ​KJD treated tibia compared to the OA tibia at follow-up (*p* ​= ​0.004; ES: 1.92)). The OARSI subscores, “PG” (*p* ​= ​0.002; ES (HG): 5.1) and “collagen pathology” (*p* ​= ​0.11, ES (HG): 1.35) were worse in the OA ​+ ​KJD femur compared to OA controls directly after KJD (Fig. 3B). At follow-up, these subscores in the OA ​+ ​KJD femur improved compared to directly after KJD (*p* ​= ​0.001; ES (HG): 2.6, and *p* ​= ​0.13; ES (HG): 0.88, respectively), although the “PG” subscore was still worse compared to the OA controls at follow-up (*p* ​= ​0.02; ES (HG): 0.97) (Fig. 3B). Interestingly, the “chondrocyte pathology” subscore was increased in the OA ​+ ​KJD tibia at follow-up compared to OA controls (*p* ​= ​0.002; ES (HG): 1.7) and compared to the OA ​+ ​KJD tibia directly after KJD (*p* ​= ​0.04; ES (HG): 1.1). This increase was mainly related to an increase in the “chondrocyte cluster formation” subscore (Fig. 3B).

### 3.5 Biochemical evaluation of cartilage alterations

**Fig. 4:**
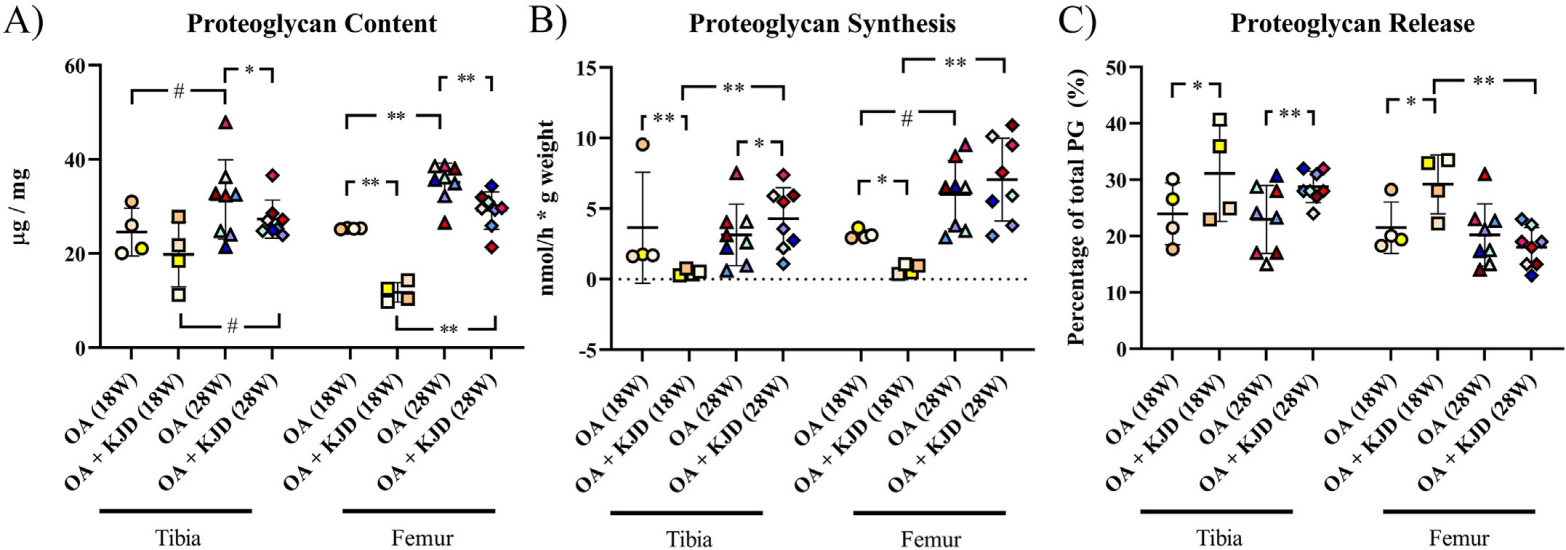
**Biochemical analysis of proteoglycans (PG) (A)** The proteoglycan (PG) content was measured in μg PG and corrected for the weight of the cartilage (mg) **(B)** The PG synthesis was measured in nmol per hour (h) and corrected for the weight of the cartilage (grams) **(C)** The PG release is the percentage (%) of Proteoglycans that is released in the medium in the 3 day culture time of the total amount of Proteoglycans. Individual coloured dots represent individual donors. ∗∗p ​< ​0.01; ∗p ​< ​0.05; # 0.15 ​> ​p ​> ​0.05 with a large ES.

Biochemical outcomes of the cartilage were in line with its histological evaluation. Directly after KJD treatment, PG content was decreased in the OA ​+ ​KJD femur (*p* ​< ​0.001; ES (HG): 5.87) compared to OA controls (Fig. 4A). This difference in PG content was lost, as the tibia and femur revealed higher PG-content in OA ​+ ​KJD joints at follow-up compared to OA ​+ ​KJD joints directly after KJD (tibia; *p* ​= ​0.09, ES (HG): 1.36, femur; *p* ​< ​0.001; ES (HG): 4.57). However, this improved PG content was in the femur still lower compared to OA controls (*p* ​= ​0.001; ES (HG): 1.4).

A similar pattern was seen for PG synthesis: it was decreased directly after KJD in the OA ​+ ​KJD tibia (*p* ​= ​0.0001, ES (HG): 0.99) and femur (*p* ​= ​0.02, ES (HG): 6.3) compared to their OA control (Fig. 4B). At follow-up, PG synthesis increased in the OA ​+ ​KJD tibia (*p* ​= ​0.001, ES (HG): 1.91) and femur (*p* ​= ​0.001, ES (HG): 2.38) compared to directly after KJD. In the tibia, this increased PG synthesis of the OA ​+ ​KJD joints at follow-up was even higher compared to OA controls (*p* ​= ​0.047, ES (HG): 0.5).

PG release, expressed as the percentage of Proteoglycans released in the medium (and normalized to the PG content), was increased in the OA ​+ ​KJD tibia and femur directly after KJD compared to OA controls (tibia; *p* ​= ​0.02; ES (HG): 0.87, femur; *p* ​= ​0.03, ES (HG): 1.37) (Fig. 4C). At follow-up, PG release decreased in the OA ​+ ​KJD femur compared to directly after KJD (p ​= ​0.001, ES (HG): 2.54) becoming comparable to the PG release of OA joints.

### 3.6 RT-qPCR analysis of the TGF-β and Notch signalling pathways

**Fig. 5:**
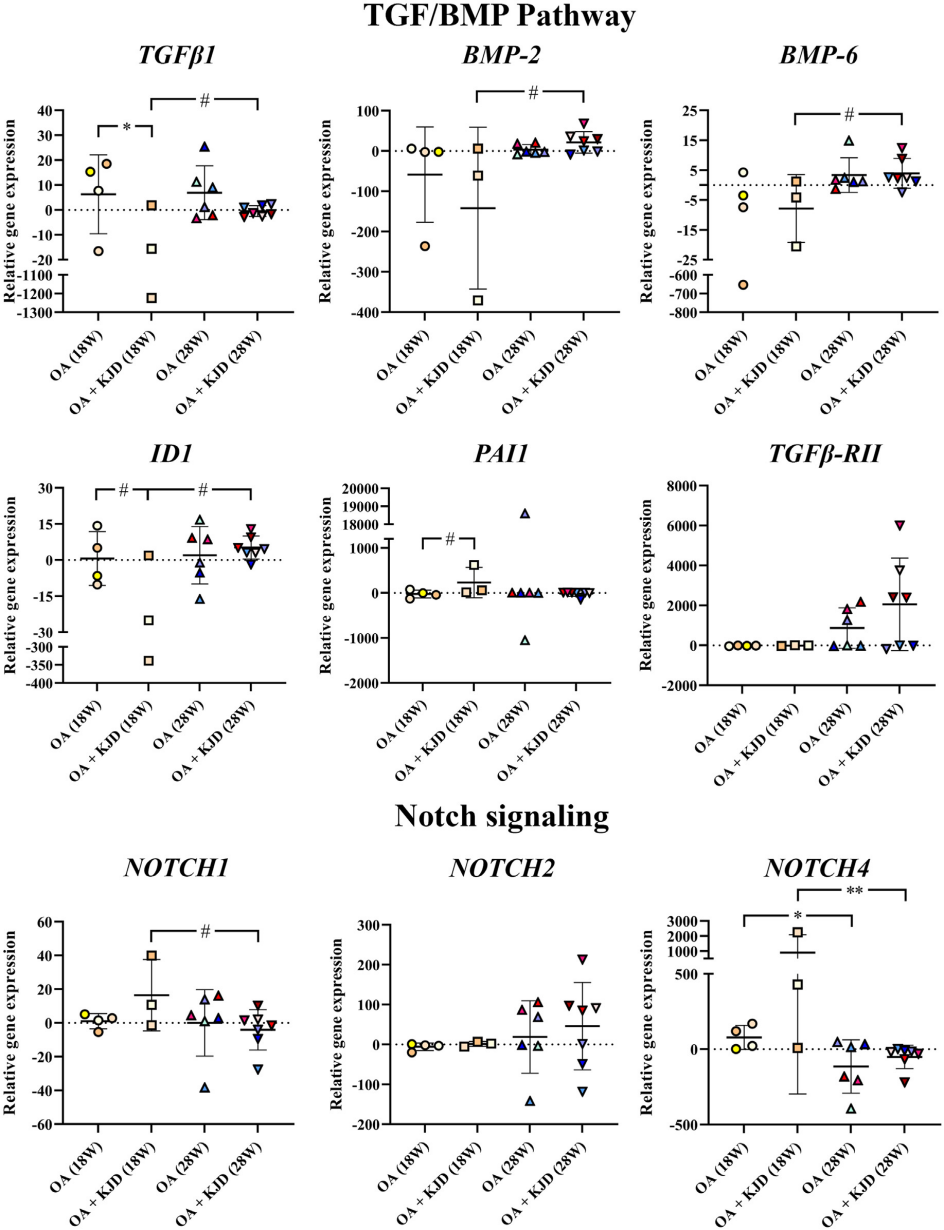
**Gene expression analysis of the TGF and Notch pathway in the cartilage.** The expression of *TGF-β1*, *BMP-2*, *BMP-6*, *ID1*, *PAI1*, and *TGF-β-RII* of the TGF pathway, and *Notch -1,-2*, and *-4* of the Notch signalling pathway is shown as the relative gene expression compared to the mean of all samples within a gene. Gene expression analysis was performed at 18 and 28 weeks for the osteoarthritic (OA), and OA treated with knee joint distraction (OA ​+ ​KJD) joints. As the bone (tibia or femur) was found to not be a determining factor in the statistical analysis, the shown expression represents the gene expression of the whole joint. The Individual coloured dots represent individual donors. ∗∗p ​< ​0.01; ∗p ​< ​0.05; # 0.15 ​> ​p ​> ​0.05 with a large ES.

RT-qPCR focused on OA-related transforming growth factor β (TGF-β) and Notch signalling pathways (Fig. 5) previously shown to be regulated during KJD [13]. Directly after KJD, in the OA ​+ ​KJD joints compared to OA controls *TGFβ1* expression was decreased (*p* ​= ​0.03; ES (HG): 1.12). In parallel *PAI1*, a well-defined downstream target of TGFβ signalling [17], was increased (*p* ​= ​0.12; ES (HG): 1.51), while *ID1*, a downstream target gene of BMP signalling, was decreased (*p* ​= ​0.12; ES (HG): 0.84). At follow-up, *TGFβ1* was increased in OA ​+ ​KJD joints compared to OA ​+ ​KJD joints directly after KJD (*p* ​= ​0.10; ES (HG): 1.21). Furthermore, in OA ​+ ​KJD joints bone morphogenetic protein 2 (*BMP-2*) (*p* ​= ​0.10, ES (HG): 1.38), *BMP-6* (*p* ​= ​0.10, ES (HG): 1.7) and *ID1* (*p* ​= ​0.10, ES (HG): 1.93) increased at follow-up compared to directly after KJD. In OA ​+ ​KJD joints, *Notch1,* a cartilage progenitor marker, was increased directly after KJD (*p* ​= ​0.14, ES (HG): 1.16) compared to follow-up. *Notch4*, upregulated in human OA joints [18], decreased significantly over time in OA joints (*p* ​= ​0.03, ES (HG): 1.39) and OA ​+ ​KJD joints (*p* ​= ​0.003, ES (HG): 3.49).

### 3.7 Evaluation of subchondral bone alterations

**Fig. 6:**
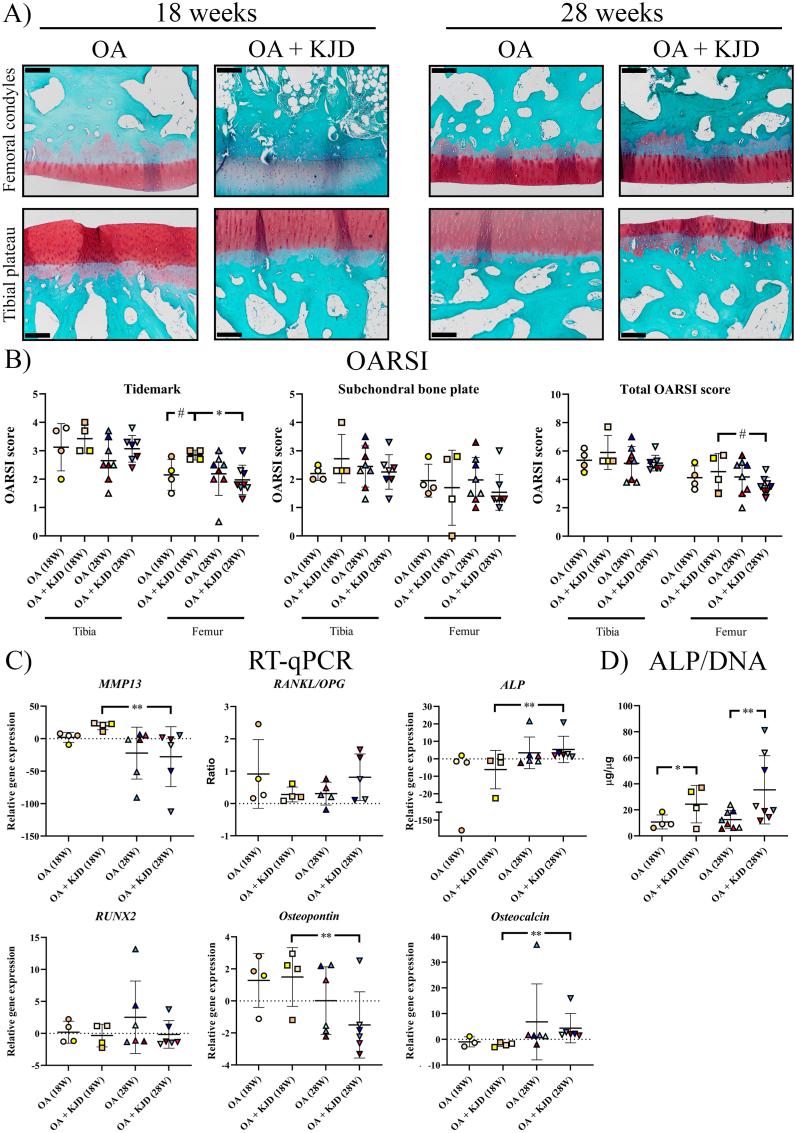
**Analysis of bone alterations**. Subchondral bone samples were collected from the femoral condyles and tibial plateaus after the cartilage was collected resulting in an osteochondral bone piece with a variable layer of cartilage. Subchondral bone sections were stained with Safranin-O/Fast green, randomized and scored blindly according to the OARSI scoring system by three observers and subsequently averaged **(A)** Representative images are shown for the osteoarthritic (OA) and OA treated with knee joint distraction (OA ​+ ​KJD) joints at 18 and 28 weeks for the femoral condyles and tibial plateaus. Scale bar is set at 100 ​μM ​**(B)** The OARSI score of the Tidemark integrity and subchondral bone plate changes are shown for all conditions **(C)** The expression of *MMP13*, *ALP*, *RUNX2*, *Osteopontin*, and *Osteocalcin* is shown as the relative gene expression compared to the mean of all samples within a gene. Gene expression analysis was performed at 18 and 28 weeks for the osteoarthritic (OA), and OA treated with knee joint distraction (OA ​+ ​KJD) joints. As the bone (tibia or femur) was found to not be a determining factor in the statistical analysis, the shown expression represents the gene expression of the whole joint. For *RANKL* and *OPG*, the *RANKL/OPG* ratio is provided for the relative gene expression of both genes **(D)** Alkaline Phosphatase (ALP) was measured in subchondral bone samples and normalized for the amount of DNA (μg/μg). Individual coloured dots represent individual donors. ∗∗p ​< ​0.01; ∗p ​< ​0.05; # 0.15 ​> ​p ​> ​0.05 with a large ES. (For interpretation of the references to colour in this figure legend, the reader is referred to the Web version of this article.)

Subchondral bone samples contained also the deep cartilage layer. OARSI scoring of Safranin-O/Fast green stained sections showed substantial variations in tidemark integrity and subchondral bone thickness scores (Fig. 6A and B). Directly after KJD, the “tidemark integrity” subscore of the OA ​+ ​KJD femur was increased compared to OA controls (*p* ​= ​0.13, ES (HG): 1.38) and compared to OA ​+ ​KJD joints at follow-up (*p* ​= ​0.03, ES (HG): 1.78), the latter resulting in an increased total OARSI score of the OA ​+ ​KJD femur at follow-up (*p* ​= ​0.13, ES (HG): 1.07) (Fig. 6B).

In order to further assist in interpretation of the histological scoring which was limited by the measured variability and evaluation in a 2D plane, ALP activity was measured, and gene expression analysis was conducted (Fig. 6C and D). The ALP/DNA concentration was increased in OA ​+ ​KJD joints compared to OA controls directly after KJD (*p* ​= ​0.02; ES (HG): 1.41) and at follow-up (*p* ​< ​0.001; ES (HG): 1.25). *Osteocalcin* and *ALP* expression, as a measure of bone formation and remodelling, were increased in OA ​+ ​KJD joints at follow-up compared to OA ​+ ​KJD joints directly after KJD (*Osteocalcin; p* ​< ​0.001; ES (CD): 0.978, *ALP; p* ​= ​0.001; ES (CD): 0.91), while the expression of *osteopontin* was decreased (*p* ​= ​0.002; ES (CD): 0.80). *MMP13* gene expression, as a measure of collagen degradation, was decreased in OA ​+ ​KJD joints at follow-up compared to OA ​+ ​KJD joints directly after KJD (*p* ​< ​0.001; ES (CD): 1.0).

### 3.8 Microscopic evaluation of synovial inflammation and synovial fluid analysis

**Fig. 7:**
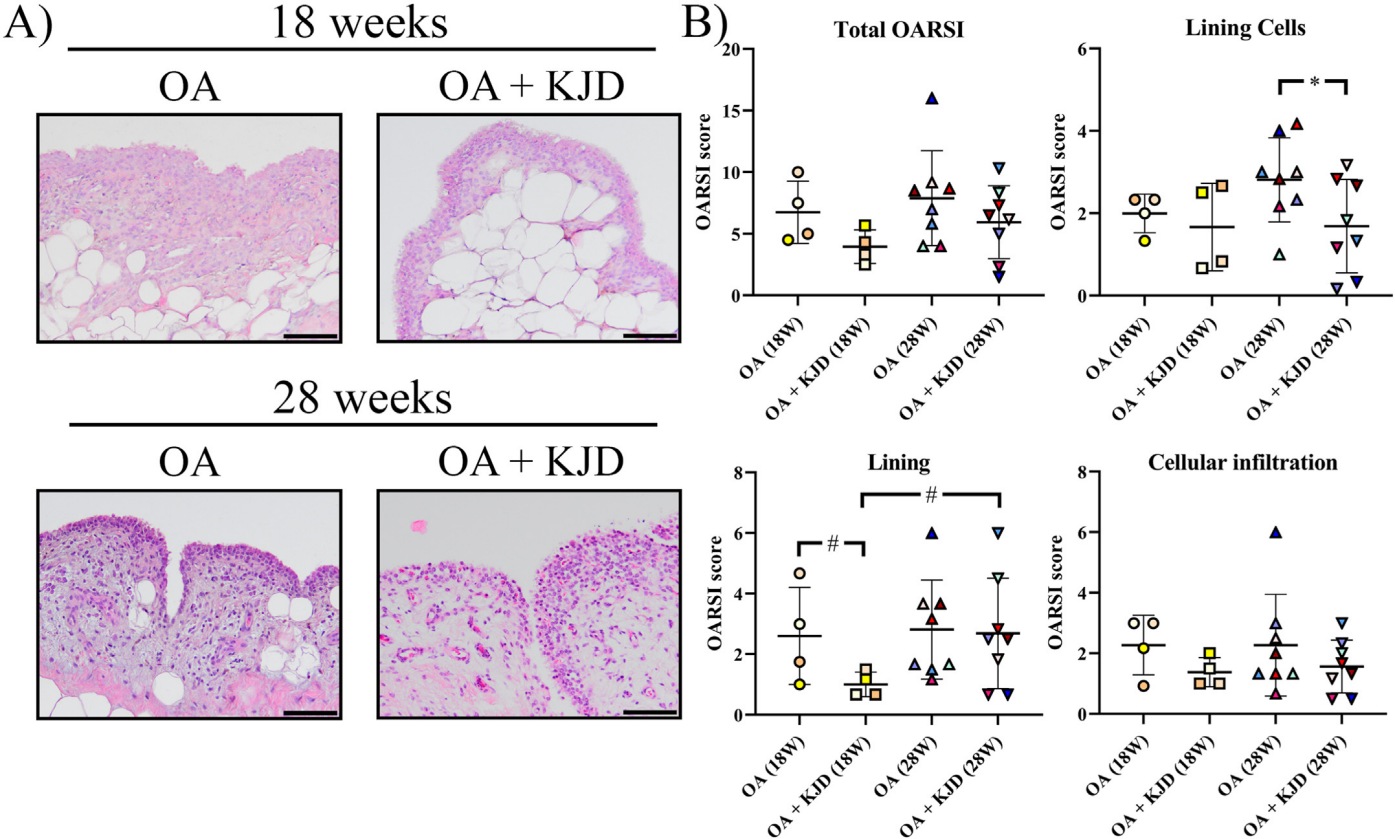
**Microscopic evaluation of synovial inflammation.** Synovial tissue sections were stained with hematoxylin-eosin (HE), randomized and scored blindly according to the OARSI scoring system by three observers and subsequently averaged **(A)** Representative images are shown for the osteoarthritic (OA) and OA treated with knee joint distraction (OA ​+ ​KJD) joints at 18 and 28 weeks. **(B)** The total OARSI score consisted of the combined score of the lining cells, lining and cellular infiltration characteristic scores. Individual coloured dots represent individual donors.

HE-stained synovial sections were evaluated using the OARSI scoring system (Fig. 7A and B). Directly after KJD, the “Lining characteristics” subscore was lower in OA ​+ ​KJD joints compared to OA controls (*p* ​= ​0.08; ES (HG): 1.19) and OA ​+ ​KJD joints at follow-up (*p* ​= ​0.06; ES (HG): 1.01) (Fig. 7B). The number of cells in the lining, “Lining Cells” subscore, was increased at follow-up in OA controls compared to OA ​+ ​KJD joints (*p* ​= ​0.04; ES (HG): 0.99).

**Fig. 8:**
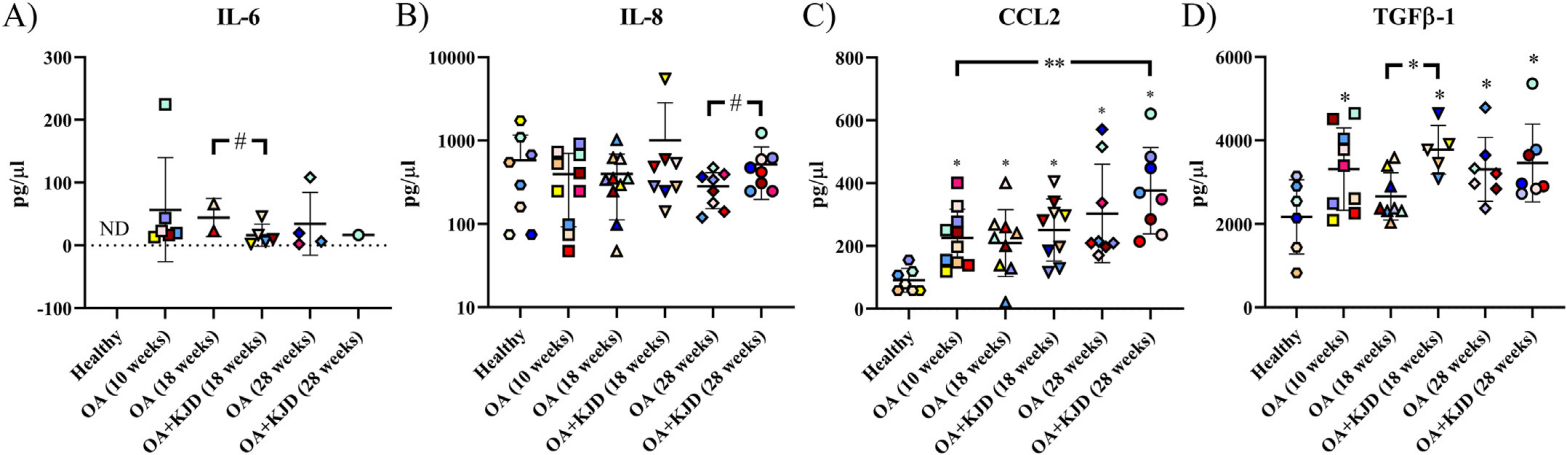
**Synovial fluid analysis of cytokines and chemokines. IL-6 (A), IL-8 (B), CCL2 (C), and TGFβ1 (D)** were measured in the synovial fluid at baseline from healthy joints (healthy), after 10 weeks of osteoarthritis induction (OA (10 weeks)), directly after treatment with knee joint distraction (KJD) at 18 weeks in the OA control (OA) and OA ​+ ​KJD joint and at 28 weeks in the OA and OA ​+ ​KJD joint. Samples were successfully obtained from 8/12 healthy baseline, 10/12 OA (10 weeks), 10/12 OA (directly after KJD (18 ​W)), 9/12 OA ​+ ​KJD (directly after KJD (18 ​W)), 8/8 OA (follow-up (28 ​W)), and 8/8 OA ​+ ​KJD (follow-up (28 ​W)) joints. ND: Not detected. ∗∗p ​< ​0.01; ∗p ​< ​0.05; # 0.15 ​> ​p ​> ​0.05 with a large ES. ∗ directly above value: *p* ​< ​0.05 compared to the healthy baseline samples. Individual coloured dots represent individual donors.

Of the cytokines and chemokines measured by the Multiplex ELISA, only IL-6, IL-8 and CCL2 were detected in >50% of the SF samples. IL-6 was undetectable in the healthy baseline SF samples. Directly after KJD, IL-6 levels were lower in OA ​+ ​KJD compared to OA SF (*p* ​= ​0.086, ES (CD): 0.8) (Fig. 8A). At follow-up, IL-8 was higher in OA ​+ ​KJD compared to OA SF (*p* ​= ​0.13, ES (HG): 0.91) (Fig. 8B). At all timepoints, CCL2 levels of the OA and OA ​+ ​KJD SF was higher compared to healthy baseline samples (*p* ​< ​0.05) (Fig. 8C). The highest CCL2 levels were found in OA ​+ ​KJD joints at follow-up, being higher compared to the healthy baseline (*p* ​< ​0.001, ES (HG): 2.69) and OA (10 weeks) SF (*p* ​= ​0.007, ES (HG): 1.26). The concentration of TGFβ1 increased after OA induction (p ​= ​0.012, ES (HG): 1.13) compared to the healthy baseline. Directly after KJD treatment, TGFβ1 levels were higher in OA ​+ ​KJD compared to the OA control at the same timepoint (p ​= ​0.022, ES (HG): 1.81).

## 4 Discussion

The present explorative study describes the dynamical changes at the cartilage, subchondral and synovial level that are associated with KJD and contributes to current concepts of how KJD elicits a reparative response seen on the long term [1].

At the structural level, the present study observed decreased PG and COL2 content directly after KJD which is in line with recent findings showing loss of PG and COL2 in the OA ​+ ​KJD joint midway distraction treatment [13]. Similar findings were also reported directly after KJD in a canine ACLT OA model [11]. This PG depletion is caused by a combination of decreased PG synthesis and increased release of PGs in the presence of increased matrix degrading enzymes, such as MMPs and ADAMTS5 [13]. These changes are most probably induced by the absence of normal loading during the KJD period. Unloading due to immobilisation or continuous joint distraction was shown to decrease PG content and synthesis in the healthy cartilage [[19], [20], [21], [22], [23]]. In addition, modest changes in the collagenous matrix of the immobilised cartilage have been reported, with a ∼13% reduction of collagen crosslinking [23]. In line with this thought, the loss of PG and COL2 content seen directly after KJD, is partially reversed after 10 weeks of follow-up where the animals are allowed to load their joints, and is mostly due to an increased PG synthesis. Correspondingly, cyclic compression increases PG synthesis in cartilage explants [24] and remobilization after joint immobilization leads to (partial) recovery of the cartilage PG content [21,23].

These observations raise the question how these changes in the cartilaginous matrix, seen during and after KJD, ultimately lead to the cartilage regenerative responses seen at prolonged follow-up in the canine experimental study [12], the small animal studies [[5], [6], [7], [8]], and the clinical studies in humans [2]. During OA, a vicious circle occurs, starting with the deterioration of the cartilaginous matrix, resulting in PG loss, increased collagen crosslinking and altered molecular organization [25]. As the mechanoprotective properties of the degenerate cartilage are lost, normal loading of the joint results in OA progression [21]. This circle is further maintained by the limited intrinsic reparative capacity of cartilage [26,27]. During KJD, the mechanical loading of the joint is decreased, causing further matrix degradation and advancement of the tidemark [21,28]. Under these conditions, it is tempting to hypothesize that this matrix degradation facilitates KJD-related repair in two manners: (a) improper matrix components due to OA are removed [29], and (2) sequestered heparan sulfate-bound molecules of the pericellular matrix, such as TGFβ1 and fibroblast growth factor 2 (FGF2) are released [21,29]. These biomolecules have both been found to be upregulated in the SF of human patients during KJD treatment [30] and in the present study TGFβ1 was upregulated in the OA ​+ ​KJD directly after KJD treatment compared to the OA control. Activated TGFβ signalling is also reflected in the increased expression of *PAI1* at the cartilage level, a downstream mediator of TGFβ1 signalling, in KJD-treated joints compared to OA controls directly after treatment, even though *TGFβ1* mRNA levels were downregulated. These changes indicated that the altered TGF signaling may contribute to the restoration to the joint homeostasis. Release of these biomolecules through matrix degradation could also induce pathways related to cartilage reparative responses. Altogether, this implies that the (partial) unloading during KJD initiates a catabolic environment, allowing for essential remodeling of the cartilage. Combined with the return of the loading after the KJD, these changes in the cartilage homeostasis provide an anabolic environment that promotes the subsequent successful cartilage repair activity.

In parallel to the matrix changes, increased chondrocyte cluster formation in the KJD-treated joints at follow-up was observed. Traditionally, an increase in cluster formation is recognized as a hallmark of OA [16]. However, chondrocyte clusters produce anabolic factors and express progenitor cell markers [31]. Indeed, both during [13] and directly after KJD treatment, *Notch1*, a cartilage progenitor cell marker [32], was upregulated in KJD-treated joints compared to OA controls. In line with this hypothesis, the released FGF2 and TGFβ1 due to the increased matrix remodelling could facilitate chondrocyte progenitor activation, proliferation and subsequent differentiation [21,29,33].

The effects of decreased loading and remobilisation upon removal of the KJD are also reflected at the subchondral bone level. This is corroborated by the changes in the tidemark integrity [28] and the increased (subchondral) bone remodelling at follow-up, as demonstrated by an increase in *ALP* and *osteocalcin*, indicating the presence of active osteoblasts [34]. The increased bone remodelling corresponds with the effects of distraction on the ankle joint in human patients in which a decreased subchondral bone density was found in sclerotic areas and an increased density in cystic lesions, resulting in an overall normalization of bone density after distraction treatment [35]. This increase in bone density in the cystic lesions might explain the decrease in pain in patients treated with distraction, as subchondral bone cysts are associated with nerve ingrowth and pain [36]. Furthermore, the effect of mechanical loading on bone is well known; once the mechanical load changes, the turnover rate of subchondral bone adjusts to adapt [37]. Notably, the insertion of the bone pins, without actual distraction, had already cartilage regenerative effects on the OA cartilage of dogs [12]. As the existence of direct molecular signalling linking cartilage and subchondral bone has been proven, the effect of this crosstalk during KJD should not be excluded [37].

The OA environment in the canine grooved joint, is reflected in the mild synovitis score on histology and the upregulated CCL2 and IL-6 SF levels in OA and KJD-treated joints compared to their healthy baseline [38]. Directly after KJD synovitis seemed to be improved based on reduced villi formation and reduced SF IL-6 levels compared to OA controls. However, there was substantial variation in IL-6 levels in the KJD joints and IL-6 levels were in only two samples detectable in the OA joint. Therefore, these results should be interpreted with caution. Interestingly, at follow-up synovitis increased again evidenced at the macroscopic and microscopic level and the increased SF IL-8 and CCL2 levels. In line with this, *Watt* et al. *(2020),* reported increased CCL2 SF levels directly after-, and increased IL-8 SF levels during KJD treatment [30]. In general, increased CCL2 and IL-8 levels are associated with OA severity [39]. The question remains however whether the synovitis found in the follow-up contributes to or is a reflection of the degenerative cartilage changes. A more detailed investigation into the phenotypic changes of the synoviocytes could provide clues into the role of the synovial membrane during KJD.

The small number of animals resulted in a high variance of parameters such as the gene expression analysis, compromising the study power of these results. Therefore, the obtained insights require further in dept investigation with a larger sample size and results should be interpreted carefully. Furthermore, technical problems arose in this study such as the high bone pin failure rate. Although no problems in the bone pin material could be identified, to prevent this future studies using the canine KJD model should reconsider the stiffness of the construction.

Joint distraction provides a biochemical and biomechanical environment that facilitates regeneration of the joint. However, it is an demanding treatment with 6–9 weeks of partial joint immobilization, and entails a high risk of skin pin-tract infections [1]. More importantly, for a group of people, joint distraction is only a temporary solution that delays the OA progression, with a clear influence of patient characteristics on the overall effect [40]. This study contributes to further elucidation of the mechanisms behind joint distraction. This could help improve the existing KJD treatment or help with patient selection. For example, based on the results of this study it could be hypothesized that patients with osteoarthritis characterized by cartilage damage or altered cartilage and bone metabolism might benefit more from KJD treatment than patients with OA characterized by chronic pain [41]. Furthermore, these insights into joint and cartilage regeneration might result in the discovery or improvement of disease modifying OA drugs (DMOADs) or other cartilage regenerative approaches [42]. These treatments could also be combined with KJD treatment. However, as we found in this study that the effect of KJD treatment consists of a catabolic and anabolic phase, it is important to consider the mode of action of the applied DMOAD and the right time frame for its application. Finally, as the dog is not only a suitable animal model for OA but also a species that suffers from spontaneous OA [10], the techniques optimized for this animal model could be applied to treat dogs with OA. KJD has been shown to be a feasible treatment strategy for dogs with severe end-stage OA, although efficacy has to be proven in larger clinical studies [15].

## 5 Conclusions

During KJD there is a catabolic environment, characterized by a loss of PG and COL2 content and a decrease in PG synthesis. This preceding cartilage degradation may remove the deteriorated osteoarthritic matrix and free growth factors sequestered in the matrix. Upon restoration of joint loading, at 10 weeks of follow-up, PG and COL2 content and PG synthesis increase, demonstrating a switch towards an anabolic joint environment. Gene expression analysis of the cartilage suggests the involvement of the TGF and Notch signalling pathways. Concurrent subchondral bone remodelling may contribute to the regenerative effects of KJD.

## Funding/support statement

This project was financially supported by the Dutch Arthritis Society (LLP9 and LLP22) and NWO Domain Applied and Engineering Sciences (AES) (P15-23). There is no further involvement in the present work of the abovementioned sources.

## Author statement

BPM, FPJGL, MAT and SCM obtained the funding for this study. MT, BPM, FB, FPJGL, MAT and SCM contributed to the conception and design of the study. MT, BPM, LS, KC, JPC, ISL, FPJGL, MAT and SCM contributed to the experimental procedures, and collection and assembly of the data. LS, KC and ISL provided technical and logistic support during the study. MT, BPM, FPJGL, MAT and SCM contributed to the analysis and interpretation of the data. All authors contributed to drafting of the article and helped revising it critically for intellectual content. All authors approved the final version of this article for submission.

## Declaration of competing interest

FPJGL is consultant as UMCU employee for Synerkine Pharma BV and co-founder of ArthroSave BV without further relations. All authors declare that there is no conflict of interest.
