# Supplementary material for: The catabolic-to-anabolic shift seen in the canine osteoarthritic cartilage treated with knee joint distraction occurs after the distraction period
Source: J Orthop Translat. 2022 Oct 20;38:44–55. doi: 10.1016/j.jot.2022.09.003 (PMC9589009; doi:10.1016/j.jot.2022.09.003)
Supplement: Multimedia component 1 [file mmc1.docx]

**Supplementary Material**

**Table of Contents**

[**1. Extended Methods and Materials** 1](#_Toc77191421)

[**1.1 Animals** 1](#_Toc77191422)

[**1.2 Experimental procedures** 2](#_Toc77191423)

[*1.2.1. Induction of OA* 2](#_Toc77191424)

[*1.2.2. Joint distraction* 4](#_Toc77191425)

[**1.3. Outcome measures** 6](#_Toc77191426)

[*1.3.1. Assessment of cartilage integrity* 6](#_Toc77191427)

[*1.3.2. Assessment of proteoglycan turnover* 6](#_Toc77191428)

[*1.3.3. Gene expression analysis by RT-qPCR* 7](#_Toc77191429)

[*1.3.4. DNA and alkaline phosphatase measurement* 8](#_Toc77191430)

[**1.4. Statistical analysis** 8](#_Toc77191431)

[**2. Adverse effects** 9](#_Toc77191432)

[**3. Sensitivity analysis** 10](#_Toc77191433)

[**4. Supplementary Tables** 11](#_Toc77191434)

[**4.1. Supplementary Table 1** 11](#_Toc77191435)

[**4.2. Supplementary Table 2** 13](#_Toc77191436)

# **1. Extended Methods and Materials**

## **1.1 Animals**

Skeletally mature mixed‐breed dogs (n = 12, females, with a mean ± SD age of 16.1±5.1 months and a weight of 20.9±2.6 kg) were obtained from Marshall BioResources (North Rose, New York, United States) after ethical approval of the National Central Committee for Animal Experiments (AVD1080020173964); the working protocols were overseen by the local Welfare Body.

All animals were housed in pairs in indoor pens (3x4 m^2^) on corn cob bedding under standard environmental conditions. They were allowed to exercise outdoors for at least 2 hours a day on a large patio (15x3.5 m^2^) during the whole experiment. Cage enrichment was provided in the form of their cage mate and toys. Throughout the experiment they were fed a standard diet and ad libitum water.

On intake, all animals received a clinical and orthopaedic examination. A standard welfare assessment was performed at least one time a week.

A sample size calculation was performed, using the size effects of the data of the earlier KJD study^1^, to calculate the number of animals needed for the experiment. For this purpose, a two-way fixed effects ANOVA was performed, using a power of 0.85 and an α of 0.0125 (corrected for the amount of comparisons) (G*power software 3.1.9.4), with the conclusion that at least 4 animals per group were needed to reach sufficient power.

Due to logistical reasons, the experiment was first performed in 8 dogs. These dogs were randomly divided in two groups at day 0. The first group (n=4) was euthanized directly after the KJD procedure was finished (Direct after KJD, 18W). The second group (n=4) was euthanized after a follow-up period of 10 weeks after the end of the KJD procedure (Follow-up, 28W). As the second time point (10 weeks follow-up) proved to be the most interesting time point, this experiment was repeated with the remaining dogs (n=4) (Figure 1).

During the study, the order in which force plate measurements, blood and synovial fluid collection, and experimental procedures were performed in the animals was alternated and randomized.

## **1.2 Experimental procedures**

*1.2.1. Induction of OA*In all animals, OA was induced bilaterally in the stifle joint according to the canine groove model.^2,3^ The bilateral OA model was used to provide an internal animal OA control and reduce the amount of animals needed, maintaining statistical power.^2^ This model was demonstrated tot show consistent and clear development of bilateral OA features, and was found to be slightly more severe compared to the unilateral groove model.^2^

Before all procedures, the dogs were put under general anaesthesia (premedication; dexmedetomidine (IM) and butorphanol (IM), induction; propofol (IV)) and received pre-emptive analgesia (buprenorphine (IV) and carprofen (IV)) and antibiotic treatment (cefazoline (IV)). All procedures were performed under sterile conditions and with continuous surveillance of vital signs (heart rate and respiration monitoring, and oxygen saturation measurements). Dogs were intubated and anesthesia was maintained by isoflurane (1.0-1.5%, inhalation).

The joint was approached using a medial mini-arthrotomy close to and parallel with the ligamentum patellae. With the joint in maximum flexion, ten grooves were made in the cartilage of the lateral and medial condyles with a Kirschner-wire (1.5-mm diameter) bent 0.4 mm from the top at 90° (ensuring that the depth of the grooves was restricted to the cartilage depth and not to the subchondral bone). Menisci and tibial plateaus were left untouched. Synovial tissue, joint capsule, and skin were sutured according to their anatomical layers.

Burprenorphine (IM) and carprofen (IM) were used peri-operative for pain management. Oral carprofen was provided for three days after surgery for additional pain management. To support optimal wound closure, dogs were kept apart and without bedding until the wound was closed, which took 2-3 days. After 24 hours, the dogs were allowed to go outside on the patio on a daily base again. During the first week, welfare of the dogs was assessed daily. During the OA induction phase, all dogs were fully active with subjectively normal joint loading and movement.

### *1.2.2. Joint distraction*

After 10 weeks of OA induction, all dogs received joint distraction treatment to the right hindlimb for 8 weeks by use of an external fixation frame with a hinge bridging the joint.^4^

To place the external fixation frame, three bone pins (3 mm in diameter; Stryker) were manually drilled into the femur; 2 distally (medial and lateral side) and 1 proximally on the craniolateral side. Additionally, three bone pins were drilled into the tibia, two proximally (medial and lateral side) and one distally on the craniomedial side. External fixation frames were custom-made and adapted to the dog’s anatomy. On both the tibia and the femur, a frame (5mm diameter rod) was connected to the bone-pins in a three-point fixation with use of commercially available connectors (Delta coupling, Stryker®). Subsequently, the external fixation frames on the femur and the tibia were connected by hinges medially and laterally of the knee joint. Distraction of the joint was carried out by extending the connecting rods and was visualized by fluoroscopy using a C-arm (Figure 1B), while smooth motion of the joint during flexion and extension was maintained.

Pain management was similar peri- and post-operatively as described above. In addition, oral antibiotic treatment (amoxicillin and clavulanic acid (Synulox; 12.5 mg/kg, Zoetis)) was provided during the first 5 days, and repeated in case of pin tract infection, as was the case in 2/12 dogs. After 2-3 days of recovery, all dogs were given access to the patio again but were housed individually for the entire distraction period. Welfare was assessed daily during the first week, including temperature measurements.

During the distraction period, dogs were less active compared to before the distraction, as assessed by the animal technicians and responsible researchers. The distracted legs were used variably during walking, but were not loaded during running. Joint distraction was monitored every two weeks by radiography in a loaded position (standing). Adjustments were made to the distraction frame in 2/12 dogs. In these dogs, the distraction was increased by extending the connecting rods while the animal was awake, until the joint space width was increased on the radiographs. Additionally, it was checked if all bone pins were intact. In 5/12 dogs, one of the femur pens broke during the distraction period. In these dogs, a new bone pin was drilled during general anesthesia, the frame was reattached and joint distraction was restored. Peri- and post-operative care was the same as described above.

After 8 weeks of KJD, the 4 dogs in the OA+KJD (18W) group were sedated and subsequently euthanized by an intravenous injection of pentobarbital (Euthanimal 20%; Alfasan Nederland B.V). At the same timepoint, the distraction frame of the dogs in the OA+KJD (28W) group (n=8) was removed in two phases in order to force the dogs to gradually reload the joint surfaces. First, the distraction was ended by removing the connecting rods and hinges. The fixation frames and bone pins were removed two weeks later under general anesthesia (Fig. 1A).

In the first two weeks after removal of the bone pins, the distracted legs were still a bit stiff with a decreased range of motion during flexion of the leg. This gradually improved to the point where both legs regained full range of motion and were fully loaded. 8 weeks after removal of the bone pins, which is 10 weeks after the end of the distraction period, the dogs in the OA+KJD (28W) group were euthanized as described above.

After euthanasia, both hindlimbs were immediately removed and processed within 2 hours. High resolution photographs were obtained of the femoral condyles, tibial plateaus and suprapatellar synovial tissue after opening the stifle joint. Cartilage and subchondral bone tissue from the weight-bearing area of the femoral condyles and tibial plateaus, and patellar synovium tissue were collected and fixed in 4% phosphate-buffered formalin containing 2% sucrose (pH 7.0) for (immuno)histochemistry or snap frozen for RNA isolation. Additionally, pieces of cartilage were collected for biochemical analysis.

## **1.3. Outcome measures**

### *1.3.1. Assessment of cartilage integrity*

Macroscopic scoring of cartilage damage and synovial inflammation was performed according to the Osteoarthritis Research Society International (OARSI) canine scoring system^5^. High resolution photographs were blinded and randomized by MT and subsequently scored by two observers (KC and SCM).

After fixation, five micrometre sections of cartilage and subchondral bone were stained with Safranin-O/Fast green staining (0.125% Safranin O (S8884, Sigma), 0.4% Fast Green (F7252, Sigma) and counterstained with Weigerts haematoxylin (640495, Klinipath). Synovial membrane sections were stained with Hematoxylin/Eosin staining (Mayers haematoxylin (109249, Merck), 0.2% Eosin (115935, Merck). Sections were blinded and randomized by KC, and subsequently scored according the OARSI canine scoring system by three observers (MT, LS, SCM). Additionally, the cartilage matrix was evaluated for the immunopositivity of collagen type-1 (COL1A1), -2 (COL2A1). Immunohistochemistry was performed as described previously^1^ using COL-I mouse monoclonal antibody (0.07 µg/mL, Abcam, ab6308) and COL-II mouse monoclonal antibody (0.02 µg/mL, DSSHB, II-II6B3), respectively. Normal mouse isotype controls (IgG1 (R&D, HAF007) showed no aspecific staining. Images were collected using light microscopy (Olympus BX43, Olympus, Leiderdorp, the Netherlands).

### *1.3.2. Assessment of proteoglycan turnover*

In order to investigate the PG synthesis, the amount of pulse‐labeled PGs was determined using ^35^SO_4_^2−^ as a tracer as described previously.^24,29^ Briefly, from predefined locations of the tibial plateaus and femoral condyles, 8 cartilage samples were collected. After four hours of labeling with Na_2_^35^SO_4_ (NEX-041-H, carrier free, Dupont; Wilmington, DE) and a subsequent 3-day culture period, the cartilage pieces were digested with papain (2% (Sigma nr. P3125 (17IE/mg protein )) and the (labelled) glycosaminoglycans (GAGs) were precipitated from the papain digest of the tissue and from the culture medium by use of or cetylpyridium chloride (CPC; Sigma C-9002) or Alcian blue (Sigma A5268), respectively. Subsequently, ^35^SO_4_^2-^ -labelled GAGs were measured by liquid scintillation analysis (TriCarb, Perkin Elmer) and normalized according to the specific activity of the medium and the labeling time. The total amount of GAGs in the cartilage tissue and culture medium, precipitated with Alcian blue, were quantified spectrophotometrically according to the change in absorbance at 620 nm, using chondroitin sulfate (C4383, Sigma) as a reference. All values were normalized to the wet weight of the cartilage tissue samples (mg/g).

### *1.3.3. Gene expression analysis by RT-qPCR*

A mikro-dismembrator (B. Braun Biotech International, Germany) was used to reduce snap-frozen cartilage and subchondral bone samples to a powder using two cycles of 2000 rpm. After lysis of the homogenate with QIAzol Lysis Reagent (79306, Qiagen, Venlo, the Netherlands) and total RNA was extracted using the RNeasy Mini (subchondral bone) or Micro (cartilage) Kit (74104/ 74004, Qiagen) according to the manufacturer’s instructions including an on-column DNase step. RNA quality and quantity were measured with a Bioanalyzer (Nano-chip, Agilent Technologies, Amstelveen, the Netherlands). Subsequently, cDNA was produced using the iScriptTM cDNA Synthesis Kit (Bio-Rad, Lunteren, the Netherlands) with a similar RNA input for all samples of one tissue type, following manufacturer´s instructions.

Quantitative RT-PCR was performed using IQ SYBR Green SuperMix and a CFX384 Touch™ Real-Time PCR Detection System (Biorad) according to the manufacturer’s protocols. No template controls (NTC) were included. Perlprimer v1.1.14 was used for primer design on Ensembl annotated transcripts. The primers (Supplementary table 1) that were used were dog-specific and validated in silico using BLAST (specificity analysis) and M-fold (secondary structure). Normalization of the gene expression was performed with 7 reference genes: *RPS19, SDHA, YWHAZ, TBP, RPS5, RPL13 and HPRT*. Samples of which the average Cq of the references genes was higher than 35 were excluded, as the quality of the sample was considered to be insufficient. Relative gene expression was calculated using the Livak method [1] (2^^-ΔΔCq^) and the mean expression of all samples.

### *1.3.4. DNA and alkaline phosphatase measurement*

The homogenized subchondral bone powder was dissolved in Tris-HCl + 2% Triton (1:1) solution (3.0 M Tris-HCl (pH 9.0, 1.08386, Merck) with 2% Triton X-100 (Sigma-aldrich)). DNA was measured using the Qubit™ dsDNA BR Assay (Q33265, Thermo Fisher Scientific), according to manufacturer’s protocol. Alkaline phosphatase (ALP) was measured using a p-nitrophenyl phosphate (pNPP) assay. Reaction buffer (consisting of activity assay buffer (ABB; 3.0 M Tris-HCl, and 5 mM ZnCl_2_ + 5 mM MgCl_2_ (1:1)), and pNPP (2.78 mg/ml ABB, (71768-5G, Sigma-Aldrich)) was added to the sample, incubated for 30 min at 37°C, and measured spectrophotometrically at 405 nm absorbance. A calibration series (2-step dilution), consisting of 4-nitrophenol (pNP, 73560-25G, Sigma-Aldrich) was used to calculate the amount of ALP in the sample, normalized for DNA content.

## **1.4. Statistical analysis**

Statistical analysis was performed using R Statistics (R version 3.6.3 [2], RStudio version 1.2.5033 [3]). Normality was tested via QQ plots, histograms, and Shapiro Wilk tests. If the data were normally distributed, linear mixed models were employed. For each parameter, the selection of random effects “donor” and “location” (tibial plateaus/femoral condyles (if applicable depending on the tissue)) was chosen based on the Akaike information criterion (AIC) index. If the variable “location” was considered a significant variable for the model, an additional analysis was run on the separated data of the tibial plateaus and femoral condyles of the cartilage and subchondral bone, and the data were visualized accordingly. Additionally, normality of the residuals was assessed for each linear model. If the data was not normally distributed, a Kruskal–Wallis test, and, if significant, a subsequent Dunn’s Multiple Comparison Test was used. For the parameters with a specific research question (e.g. OARSI scores and biochemical analysis), no multiple comparison correction was used due to the explorative nature of the study. For the RT-qPCR analysis, P values were subjected to corrections for multiple testing (Benjamini-Hochberg False Discovery Rate). Effect sizes (ES) and ES’s 95% confident intervals (CI) were provided as Hedge’s g (HG) for normally distributed data and Cliff’s delta (CD) for non-normally distributed data. Because of the explorative nature of the study, outcomes with a *p* value of < 0.05 or *p* < 0.15 in combination with a large effect size (HG > 0.8, CD > 0.47) were considered relevant for discussion. For these outcomes, the mean difference (MD) with CI is provided in text.

# **2. Adverse effects**

During the OA induction phase, all dogs were fully active with subjectively normal joint loading and movement.1 After the groove surgery, 1/12 dogs developed seroma formation at the incision site of the left knee, which resolved within a few days. During KJD treatment, dogs were less physically active compared to before the distraction, as assessed by the animal technicians and responsible researchers. In 5/12 dogs, one of the femur pens broke during the distraction period. In these dogs, a new bone pin was placed under general anaesthesia, the frame was reattached, and joint distraction was restored. In 1/8 of the dogs in the 28W group, the bone pins were removed immediately after the end of the distraction period, instead of after the two week adjustment phase, due to clinical and radiological signs of osteomyelitis. Additional treatment with carprofen and antimicrobials was initiated, and the dog showed a good recovery within one week.

# **3. Sensitivity analysis**

During collection of the materials, we observed that in the OA control leg of 5/12 animals, the grooves were situated in the patellar groove instead of on the weight bearing side of the femoral condyles (Fig. 2A), resulting in an OARSI score of 0 (Fig. 2B). A sensitivity analysis was performed to investigate the effect of these findings on the data of the other parameters. For this purpose, the data of the macroscopic and microscopic OARSI scoring and the biochemical data was compared with special attention to the statistical effect and the direction of this effect (Supplementary table 2). In the end, no significant effect of the groove location was found on all parameters and all data were included in further analysis.

**Supplementary References**

[1] Schmittgen TD, Livak KJ. Analyzing real-time PCR data by the comparative CT method. Nat Protoc 2008;3:1101–8.

[2] R Core Team (2020). R: A language and environment for statistical computing. 2020.

[3] Rstudio Team. RStudio: Integrated Development for R. 2020. dttps://doi.org/10.1145/3132847.3132886.

**4. Supplementary Tables**

## **4.1. Supplementary Table 1**

**Supplementary table 1: Primers used in the RT-qPCR analysis.** Primers are divided in functional groups. F; forwards, R; reverse sequence.

| **Category** | **Gene** | **Primer Sequence** | **Annealing temperature (°C)** |
| --- | --- | --- | --- |
| **TGF/BMP pathway** | *TGFβ1* | F: CAAGGATCTGGGCTGGAAGTGGA | 69 |
|  |  | R: CCAGGACCTTGCTGTACTGCGTGT |  |
|  | *BMP-2* | F: CAGAAATGAGTGGGAAAACAAC | 64-65 |
|  |  | R: GTCTGGTCACGGGGAACTT |  |
|  | *BMP-6* | F: GCCTCAGATTACAACAGCAG | 68 |
|  |  | R: CCATCACAGTAGTTGGCG |  |
|  | *ID1* | F: CTCAACGGCGAGATCAG | 59.5 |
|  |  | R: GAGCACGGGTTCTTCTC |  |
|  | *PAI1* | F: AAACCTGGCGGACTTCTC | 61.5 |
|  |  | R: ACTGTGCCACTCTCATTCAC |  |
|  | *TGFβ1-RII* | F: GACCTGCTGCCTGTGTGACTTTG | 61 |
|  |  | R: GGACTTCGGGAGCCATGTATCTTG |  |
| **Notch pathway** | *NOTCH1* | F: TACCGGCCAGAACTGTGAGGAGAA | 54.5-60 |
|  |  | R: GGAGGGCAGCGGCAGTTGTAAGTA |  |
|  | *NOTCH2* | F: AGCACGCATCCTGGCATACCTC | 57-60 |
|  |  | R: TGGGGATTAGCTGGAAAGTCACAA |  |
|  | *NOTCH4* | F: GGAAGGGAGCCAGGGACCAACACA | 64-72 |
|  |  | R: TCAGGGCCACAGCGGGACAAATC |  |
| **Bone remodelling** | *MMP13* | F: CTGAGGAAGACTTCCAGCTT | 65 |
|  |  | R: TTGGACCACTTGAGAGTTCG |  |
|  | *RANKL* | F: AGAGCATTAAGCAGGC | 50 |
|  |  | R: TATGAGTCTTGCCCCT |  |
|  | *OPG* | F: GGGTTCTTCTCGAATGAGACG | 59 |
|  |  | R: CCTGAAGAATGCCTCCTCAC |  |
|  | *ALP* | F: GGCTTCAGAATCTCAACAC | 55 |
|  |  | R: AACTTGTCCATCTCCAGC |  |
|  | *RUNX2* | F: AACGATCTGAGATTTGTGGGC | 64 |
|  |  | R: TGTGATAGGTGGCTACTTGGG |  |
|  | *Osteopontin* | F: GAATGCTGTGCTGACTGAGG | 66-67 |
|  |  | R: TGGCTATCCACATCGTCTCC |  |
|  | *Osteocalcin* | F: CTGATGGTCCTTGCCCT | 60-63 |
|  |  | R: CTTGGACACGAAGGTTGC |  |
| **Reference genes** | *HPRT* | F: AGCTTGCTGGTGAAAAGGAC | 56-58 |
|  |  | R: TTATAGTCAAGGGCATATCC |  |
|  | *RPL13* | R: GCCGGAAGGTTGTAGTCGT | 61 |
|  |  | R: GGAGGAAGGCCAGGTAATTC |  |
|  | *RPS19* | F: CCTTCCTCAAAAAGTCTGGG | 61 |
|  |  | R: GTTCTCATCGTAGGGAGCAAG |  |
|  | *RPS5* | F: TCACTGGTGAGAACCCCCT | 62.5 |
|  |  | R: CCTGATTCACACGGCGTAG |  |
|  | *SDHA* | F: GCCTTGGATCTCTTGATGGA | 61 |
|  |  | R: TTCTTGGCTCTTATGCGATG |  |
|  | *TBP* | F: CTATTTCTTGGTGTGCATGAGG | 57 |
|  |  | R: CCTCGGCATTCAGTCTTTTC |  |
|  | *YWHAZ* | F: CGAAGTTGCTGCTGGTGA | 58 |
|  |  | R: TTGCATTTCCTTTTTGCTGA |  |

## **4.2. Supplementary Table 2**

**Supplementary table 2: Sensitivity analysis.** A sensitivity analysis was performed to investigate the effect of these findings on the data of the other parameters. The complete data set (All data included) was compared with the dataset that excluded the left hindlimbs that showed grooves in the patellar region (Patellar-grooved legs excluded). Data of the macroscopic and microscopic OARSI scoring and the biochemical data was compared for each bone (tibia or femur) and timepoint (Directly after KJD (18W) and at 10 weeks of follow-up (28W) with special attention to the statistical effect (effect), the direction of this effect, the t-, or z-value, and the *p*-value.

|  |  |  | **All data included** | | | | | |  | **Patellar-grooved legs excluded** | | | | | |
| --- | --- | --- | --- | --- | --- | --- | --- | --- | --- | --- | --- | --- | --- | --- | --- |
| **Parameter** | **Bone** | **Tijdpunt** | **Analysis type** | **Effect** | **Std. Error Effect** | **t-value** | **z-value** | ***P*-value** |  | **Analysis type** | **Effect** | **Std. Error Effect** | **t-value** | **z-value** | ***P*-value** |
| ***Macroscopy (OARSI)*** |  |  |  |  |  |  |  |  |  |  |  |  |  |  |  |
| Cartilage | Femur | 18W | Non-parametric | x | x | x | -1,21 | 0,2249 |  | Linear Mixed Model | 0,38 | 0,23 | 1,63 | x | 0,1639 |
|  |  | 28W | Non-parametric | x | x | x | -1,43 | 0,1527 |  | Linear Mixed Model | -0,18 | 0,20 | -0,91 | x | 0,4064 |
|  | Tibia | 18W | Non-parametric | x | x | x | -0,97 | 0,3329 |  | Non-parametric | x | x | x | -0,84 | 0,3982 |
|  |  | 28W | Non-parametric | x | x | x | -2,63 | 0,0085 |  | Non-parametric | x | x | x | -0,83 | 0,4077 |
| Synovium | x | 18W | Non-parametric | x | x | x | 0,28 | 0,7793 |  | Linear Mixed Model | 0,00 | 0,35 | 0,00 | x | 1,0000 |
|  |  | 28W | Non-parametric | x | x | x | -1,53 | 0,1256 |  | Linear Mixed Model | -0,49 | 0,22 | -2,21 | x | 0,0431 |
| ***Microscopy (OARSI)*** |  |  |  |  |  |  |  |  |  |  |  |  |  |  |  |
| Total OARSI | Femur | 18W | Linear Mixed Model | 2,33 | 2,22 | 1,05 | x | 0,3066 |  | Linear Mixed Model | 1,86 | 2,69 | 0,69 | x | 0,4500 |
|  |  | 28W | Linear Mixed Model | -2,40 | 1,57 | -1,52 | x | 0,1433 |  | Linear Mixed Model | -1,69 | 2,16 | -0,78 | x | 0,4464 |
|  | Tibia | 18W | Linear Mixed Model | 1,58 | 1,62 | 0,98 | x | 0,3407 |  | Linear Mixed Model | 0,97 | 1,95 | 0,50 | x | 0,6260 |
|  |  | 28W | Linear Mixed Model | -3,92 | 1,15 | -3,41 | x | 0,0027 |  | Linear Mixed Model | -4,42 | 1,57 | -2,82 | x | 0,0130 |
| Cartilage Surface | Femur | 18W | Non-parametric | x | x | x | 0,03 | 0,9799 |  | Linear Mixed Model | -0,29 | 1,09 | -0,27 | x | 0,7932 |
|  |  | 28W | Non-parametric | x | x | x | -1,08 | 0,5565 |  | Linear Mixed Model | 0,21 | 0,88 | 0,24 | x | 0,8153 |
|  | Tibia | 18W | Linear Mixed Model | -0,25 | 0,61 | -0,41 | x | 0,6874 |  | Linear Mixed Model | -0,50 | 0,71 | -0,71 | x | 0,4894 |
|  |  | 28W | Linear Mixed Model | -0,69 | 0,43 | -1,59 | x | 0,1280 |  | Linear Mixed Model | -0,54 | 0,57 | -0,96 | x | 0,3534 |
| Chondrocytes | Femur | 18W | Linear Mixed Model | -1,25 | 1,29 | -0,97 | x | 0,3455 |  | Linear Mixed Model | -1,21 | 1,50 | -0,80 | x | 0,4340 |
|  |  | 28W | Linear Mixed Model | -0,58 | 0,91 | -0,64 | x | 0,5310 |  | Linear Mixed Model | -0,58 | 1,21 | -0,48 | x | 0,6353 |
|  | Tibia | 18W | Linear Mixed Model | -0,03 | 0,42 | -0,07 | x | 0,9426 |  | Linear Mixed Model | -0,21 | 1,02 | -0,20 | x | 0,8413 |
|  |  | 28W | Linear Mixed Model | -1,30 | 0,29 | -4,43 | x | 0,0013 |  | Linear Mixed Model | -2,31 | 0,82 | -2,82 | x | 0,0129 |
| Proteoglycans | Femur | 18W | Linear Mixed Model | 0,77 | 0,22 | 3,55 | x | 0,0020 |  | Linear Mixed Model | 0,77 | 0,24 | 3,27 | x | 0,0052 |
|  |  | 28W | Linear Mixed Model | -0,38 | 0,15 | -2,48 | x | 0,0223 |  | Linear Mixed Model | -0,50 | 0,19 | -2,61 | x | 0,0196 |
|  | Tibia | 18W | Non-parametric | x | x | x | -1,63 | 0,1546 |  | Non-parametric | x | x | x | -1,37 | 0,2038 |
|  |  | 28W | Non-parametric | x | x | x | -1,76 | 0,2366 |  | Non-parametric | x | x | x | -2,33 | 0,0601 |
| ***Biochemie*** |  |  |  |  |  |  |  |  |  |  |  |  |  |  |  |
| Synthesis | Femur | 18W | Linear Mixed Model | -2,45 | 0,91 | -2,69 | x | 0,0226 |  | Linear Mixed Model | -2,52 | 1,63 | -1,55 | x | 0,1428 |
|  |  | 28W | Linear Mixed Model | -1,05 | 0,64 | -1,63 | x | 0,1336 |  | Linear Mixed Model | 0,76 | 1,31 | 0,58 | x | 0,5715 |
|  | Tibia | 18W | Linear Mixed Model | -1,76 | 0,25 | -7,00 | x | 0,0000 |  | Linear Mixed Model | -3,85 | 1,83 | -2,10 | x | 0,0527 |
|  |  | 28W | Linear Mixed Model | -0,41 | 0,18 | -2,31 | x | 0,0438 |  | Linear Mixed Model | 0,39 | 1,47 | 0,27 | x | 0,7937 |
| Content | Femur | 18W | Linear Mixed Model | -13,55 | 1,35 | -10,05 | x | 0,0000 |  | Linear Mixed Model | -14,30 | 0,96 | -14,88 | x | 0,0000 |
|  |  | 28W | Linear Mixed Model | 5,97 | 0,95 | 6,26 | x | 0,0001 |  | Linear Mixed Model | 0,23 | 0,04 | 5,60 | x | 0,0025 |
|  | Tibia | 18W | Linear Mixed Model | -4,72 | 2,25 | -2,09 | x | 0,0626 |  | Linear Mixed Model | -4,78 | 1,79 | -2,67 | x | 0,0444 |
|  |  | 28W | Linear Mixed Model | 4,17 | 1,59 | 2,62 | x | 0,0256 |  | Linear Mixed Model | 0,24 | 0,08 | 3,08 | x | 0,0274 |
